# Infection dynamics in ecosystems: on the interaction between red and grey squirrels, pox virus, pine martens and trees

**DOI:** 10.1098/rsif.2021.0551

**Published:** 2021-10-13

**Authors:** M. G. Roberts, J. A. P. Heesterbeek

**Affiliations:** ^1^ School of Natural and Computational Sciences, New Zealand Institute for Advanced Study and the Infectious Disease Research Centre, Massey University, Private Bag 102 904, North Shore Mail Centre, Auckland, New Zealand; ^2^ Department of Population Health Sciences, Faculty of Veterinary Medicine, University of Utrecht, Yalelaan 7, Utrecht 3584 CL, The Netherlands

**Keywords:** ecology, epidemiology, reservoir of infection, dilution effect, squirrel pox virus

## Abstract

Ecological and epidemiological processes and interactions influence each other, positively and negatively, directly and indirectly. The invasion potential of pathogens is influenced by the ecosystem context of their host species’ populations. This extends to the capacity of (multiple) host species to maintain their (common) pathogen and the way pathogen dynamics are influenced by changes in ecosystem composition. This paper exemplifies these interactions and consequences in a study of red and grey squirrel dynamics in the UK. Differences and changes in background habitat and trophic levels above and below the squirrel species lead to different dynamic behaviour in many subtle ways. The range of outcomes of the different interactions shows that one has to be careful when drawing conclusions about the mechanisms and processes involved in explaining observed phenomena concerning pathogens in their natural environment. The dynamic behaviour also shows that planning interventions, for example for conservation purposes, benefits from understanding the complexity of interactions beyond the particular pathogen and its threatened host species.

## 1. Introduction

The dynamics of pathogens in natural systems are shaped not only by their host species but by the entire ecosystem of which they are part [1–3]. The ecosystem can harbour a variety of host species for a given pathogen, even at different trophic levels. The dynamics of these species, and their within- and between-species interactions, provide the environment in which the pathogen survives, reproduces and spreads. There are many more species at all trophic levels that are not susceptible to a particular pathogen, but nevertheless interact with its host species. Although it can be difficult to strictly differentiate between the host and non-host species of a pathogen [4,5], for example because of varying degrees of competence [6], it is clear that a pathogen’s survival is determined in an environment containing many more interacting species than just its recognized host species. It is important to study the way ecological and epidemiological processes influence each other in order to understand the observed dynamics of a pathogen and the populations of its host species in their natural setting.

We have previously defined a general and relatively simple model eco-epidemiological system that incorporates competition within and between any number of host and non-host species, including consumer–resource relationships [7–9]. Using this system, we have shown that even subtle effects on interaction strengths between ecosystem species influence the pathogen dynamics among its hosts. We have shown that concepts such as dilution/amplification effects, maintenance species or community, reservoir species or community, that predominantly were thought of (and hence studied) in terms of host species alone, were in fact strongly influenced by interactions with non-host species [8,9]. Indeed, the ecosystem context may result in the same set of host species being a maintenance community or reservoir for a pathogen in one ecosystem, but not in another. Something similar holds for whether there is a dilution or amplification effect for a pathogen. It seems that taking a fuller view of the ecosystem context is needed to be able to understand observed patterns of pathogen dynamics and the roles of host species in those dynamics.

Here, we illustrate this need, and the explanatory power obtained when taking a fuller but still manageable ecosystem view. We study a host–pathogen system that has the minimal requirements for a meaningful analysis: a single pathogen, multiple host species, non-host species, different trophic levels, competition and consumer–resource interaction, and different observed dynamics under different circumstances. We explore the dynamics and interaction of red squirrels (*Sciurus vulgaris*) and grey squirrels (*S. carolinensis*) in different regions of the UK, with and without squirrel pox virus (SQPV). It is decidedly not our aim to present a detailed model or analysis of this system; many excellent studies exist that merge SQPV models with data to yield insight into the prospects for the red squirrel and aid conservation efforts [10–19]. Our aim is to highlight how ecological processes influence the interaction between a pathogen and its host species. We argue that analysis of a relatively well-studied system clarifies the need to take a wider ecosystem view into account when exploring dynamics involving pathogens. Our basic ingredients are a pathogen, SQPV; its host species, red and grey squirrels; their resources; and a predator. The virus is lethal for red squirrels, but grey squirrels are unaffected carriers [20–22]. Red and grey squirrels compete for food and partially for the same habitat, causing stress and pressure on red squirrels [23–27], with grey squirrels generally being the superior competitor as they use a broader range of resources. Grey squirrels are predated on by pine martens (*Martes martes*) in some of the areas where both species occur [28–30]. Over a longer period of red and grey squirrel interaction, forest management may have resulted in a gradual change in some areas towards types of trees (resources) that favour grey squirrels over red squirrels and, in turn, forest management is considered as a means to increase the competitive strength of red squirrels [31–36]. We use the insights from the cited papers as inspiration and focus on a qualitative analysis of the system dynamics. Depending on the aspects included, we study patterns in the red and grey squirrel interaction in England and Scotland, focussing on steady-state abundance levels, stability and bifurcation structure. The results illustrate the richness of analysis that can be performed and insights that can be gained when combining even rudimentary ecology and epidemiology in a toy ecosystem, and show how essential it is to recognize the indirect roles of non-host species at various trophic levels.

## 2. On eco-epidemiological dynamics involving three trophic levels

We describe the dynamics and interactions of red and grey squirrels, a predator (the pine marten), and a pathogen (SQPV) for which both squirrel species are hosts but the predator is a non-host. In addition, we consider the resources for the red and grey squirrels in terms of the habitat/food quality and availability, which may locally differentially favour one squirrel species over the other. We account for the imbalance in an abstract way, as the relative potential carrying capacities of red and grey squirrels in the absence of the alternate species. Local changes in habitat/food quality, for example as a result of forest management, are assumed to occur on a much slower time scale than the other processes and interactions in our system, and hence we do not explicitly model squirrel resource dynamics. Overall, we regard three trophic levels: the basic resources (collectively and for convenience called *trees*), the herbivore level (squirrels, SQPV) and the carnivore level (pine marten), as well as their interaction.

For the model specification and analysis, we use the approach and notation from [7–9]. Let subscripts *r* and *g* signify red and grey squirrels respectively, and subscript *p* signify the predator. The red squirrel dies from SQPV, otherwise we assume no direct effect on population dynamics. The dynamics of the host and predator species are described by

2.1 $$\begin{array}{rl} \frac{dN_{r}}{dt} & =\nu_{r}N_{r}-(\mu_{r}+\phi_{r}N_{r}+\phi_{rg}N_{g}+\psi_{rp}N_{\;p})N_{r}-\alpha I_{r}\\ \frac{dN_{g}}{dt} & =\nu_{g}N_{g}-(\mu_{g}+\phi_{gr}N_{r}+\phi_{g}N_{g}+\psi_{gp}N_{\;p})N_{g}\\ \;\mathrm{and}\;\frac{dN_{\;p}}{dt} & =(\nu_{\;p}+e_{\;pr}\psi_{rp}N_{r}+e_{\;pg}\psi_{gp}N_{g})N_{\;p}-(\mu_{\;p}+\phi_{\;p}N_{\;p})N_{\;p}, \end{array}\}$$

where *N*_*r*_, *N*_*g*_ and *N*_*p*_ are the population densities of red and grey squirrels and the predator, respectively, and *I*_*r*_ is the density of infectious red squirrels. All parameters are defined in table 1.

**Table 1.** Parameter values used in the figures.

red squirrels		
*ν*_*r*_ = 1.0 yr^−1^	maximum birth rate	[37]
*μ*_*r*_ = 0.4 yr^−1^	minimum death rate	[38]
*ϕ*_*r*_ = 0.6 yr^−1^	scaled to make *K*_*r*_ = 1	
*ϕ*_*rg*_ = 0.992 yr^−1^	competition pressure from grey squirrels	
grey squirrels		
*ν*_*g*_ = 1.2 yr^−1^	maximum birth rate	[37]
*μ*_*g*_ = 0.4 yr^−1^	minimum death rate	[38]
*ϕ*_*g*_ = 0.8 yr^−1^	scaled to make *K*_*g*_ = 1	
*ϕ*_*gr*_ = 0.32 yr^−1^	competition pressure from red squirrels	
SQPV		
*α* = 26 yr^−1^	excess death rate of infected red squirrels	[15]
*β*_*r*_ = 30 yr^−1^	infection rate, red squirrel to red squirrel	
*β*_*rg*_ = 10 yr^−1^	infection rate, grey squirrel to red squirrel	
*β*_*gr*_ = 10 yr^−1^	infection rate, red squirrel to grey squirrel	
*β*_*g*_ = 20 yr^−1^	infection rate, grey squirrel to grey squirrel	
*σ* = 13 yr^−1^	recovery rate of infected grey squirrels	[15]
*η* = 1 yr^−1^	recrudescent rate of infected grey squirrels	[12]

For simplicity, we have removed repeated subscripts, writing for example *ϕ*_*r*_ = *ϕ*_*rr*_. The parameters *ϕ* denote the within- and between-squirrel species competition for resources, the parameters *ψ* denote the strength of predation interaction (with *e* describing the conversion efficiency). The parameters *ν* and *μ* denote the birth rate and minimum death rate, respectively. For convenience, all species are assumed to show logistic growth in the absence of the other species. The carrying capacities of red and grey squirrels in the absence of the other squirrel species and the predator are *K*_*r*_ = (*ν*_*r*_ − *μ*_*r*_)/*ϕ*_*r*_ and *K*_*g*_ = (*ν*_*g*_ − *μ*_*g*_)/*ϕ*_*g*_, respectively.

We focus our analysis involving the predator on the situation in Scotland where the pine marten is present and has been increasing [29,30]. The equation for the predator in system (2.1) is given for completeness only. We assume that the pine marten is not dependent on squirrels for its population growth and that, for the time being, squirrels represent a relatively small novel food source additional to its normal resources. This translates into two assumptions: logistic growth in the absence of squirrels and a potentially much larger effect of the predator on the squirrel populations than *vice versa* (equation (2.1)). In fact, we assume that *ν*_*p*_ ≫ *e*_*pr*_*ψ*_*rp*_ *N*_*r*_ + *e*_*pg*_*ψ*_*gp*_ *N*_*g*_ and that the predator population moves rapidly to steady state $N_{\;p}^{*}$. This assumption seems reasonable in the early phases of red and grey squirrel competition in Scotland. In our analysis, the predator’s effects are then experienced as an additional rate of mortality and affect the local carrying capacity that the red and grey squirrels could attain in each other's absence. Increasing the predator steady-state abundance then allows us to traverse the landscape of qualitative dynamic behaviour of the system in terms of the carrying capacities *K*_*r*_ and *K*_*g*_, replacing *μ*_*r*_ and *μ*_*g*_ with $\mu_{r}+\psi_{rp}N_{\;p}^{*}$ and $\mu_{g}+\psi_{gp}N_{\;p}^{*}$, respectively, in the presence of the predator. In the electronic supplementary material, we present, for completeness, results for the more general case of full predator dynamics and interaction.

The dynamics of infectious red and grey squirrels are modelled by density-dependent within- and between-species transmission

2.2 $$\begin{array}{rl} \frac{dI_{r}}{dt} & =S_{r}(\beta_{r}I_{r}+\beta_{rg}I_{g})-(\mu_{r}+\alpha +\phi_{r}N_{r}+\phi_{rg}N_{g}\\ & \;+\psi_{rp}N_{\;p})I_{r}\\ \frac{dI_{g}}{dt} & =S_{g}(\beta_{gr}I_{r}+\beta_{g}I_{g})-(\mu_{g}+\sigma \\ & \;+\phi_{gr}N_{r}+\phi_{g}N_{g}+\psi_{gp}N_{\;p})I_{g}+\eta A_{g}\\ \mathrm{and}\;\frac{dA_{g}}{dt} & =\sigma I_{g}-(\mu_{g}+\eta +\phi_{gr}N_{r}+\phi_{g}N_{g}+\psi_{gp}N_{\;p})A_{g}, \end{array}\}$$

where *N*_*r*_ = *S*_*r*_ + *I*_*r*_, *N*_*g*_ = *S*_*g*_ + *I*_*g*_ + *A*_*g*_, *S*_*r*_ and *S*_*g*_ represent the population densities of susceptible red and grey squirrels, respectively. Infection with SQPV is assumed to be lifelong in red and grey squirrels, but for grey squirrels it has been shown that there may be intermittent shedding of the virus [12]. We incorporate the latter by a class *A*_*g*_ that represents the population density of grey squirrels that have been infected, but are not infectious.

We explore the dynamics of the model specified by equations (2.1) and (2.2) as follows. For a number of specific scenarios, we determine how the steady-state (equilibrium) values of the model are determined by the values of the carrying capacities *K*_*r*_ and *K*_*g*_. Having determined the possible steady states with one or both species present, without and with SQPV, we determine the stability of each steady state to small disturbances. This is achieved in two ways for infection-free steady states, by computing eigenvalues of the Jacobian matrix or the next generation matrix (NGM), and via the Jacobian matrix for infected steady states [39]. We determine thresholds for transition between stable steady states analytically from the model, and confirm these by our numerical calculations. A model of the interactions between two host species and a pathogen (unspecified) was analysed in [40]. That model had no density-dependent regulation of the infected populations, and the thresholds for transition between infected steady states were presented in terms of the host population densities rather than the reproduction numbers that we employ. The study presented in [17] addressed two squirrel species, SQPV and the pine marten as predator, but did not explicitly model infection dynamics in the grey squirrel. One feature of our model that differs from most previous models is the inclusion of the infected but not infectious state *A*_*g*_. We included this state to increase generality because it is unclear what happens to grey squirrels after recovery from SQPV. Our aim is not to fully explore the squirrel–SQPV system, but rather to more generically use the setting to highlight interactions between ecology and epidemiology. A model without this state can be recovered by setting *σ* = *η* = 0. This would not change the expressions for the thresholds presented in table 2, although the numerical values of the steady states would change. The stability results derived in electronic supplementary material would also be unchanged.

**Table 2.** Thresholds for transition between steady states. The states are labelled with the steady-state values of *N*_*r*_ and *N*_*g*_ only.

state		between-state threshold
(*K*_*r*_, 0)	$\left(N_{r}^{♭},N_{g}^{♭}\right)$	*K*_*g*_ = *B*_*r*_ = *ϕ*_*gr*_*K*_*r*_ / *ϕ*_*g*_
(0, *K*_*g*_)	$\left(N_{r}^{♭},N_{g}^{♭}\right)$	*K*_*r*_ = *B*_*g*_ = *ϕ*_*rg*_*K*_*g*_ / *ϕ*_*r*_
$\left(N_{r}^{\#},0\right)$	$\left(N_{r}^{*},N_{g}^{*}\right)$	$K_{g}=B_{r}^{\#}=\phi_{gr}N_{r}^{\#}$ / *ϕ*_g_
$\left(0,N_{g}^{\#}\right)$	$\left(N_{r}^{*},N_{g}^{*}\right)$	$K_{r}=B_{g}^{\#}=B_{g}+\alpha \beta_{rg}I_{g}^{\#}/(\phi_{r}(\phi_{rg}N_{g}^{\;}+\beta_{rg}I_{g}^{\#}+\mu_{r}+\alpha ))$
(*K*_*r*_, 0)	$\left(N_{r}^{\#},0\right)$	$R_{r}=\beta_{r}K_{r}T_{r}=1$
(0, *K*_*g*_)	$\left(0,N_{g}^{\#}\right)$	$R_{g}=\beta_{g}K_{g}T_{g}=1$
$\left(N_{r}^{♭},N_{g}^{♭}\right)$	$\left(N_{r}^{*},N_{g}^{*}\right)$	$R_{0}=\rho (K(N_{r}^{♭},N_{g}^{♭}))=1$

The system of equations (2.1) and (2.2) has multiple steady states. The Jacobian matrix is structured

$$J=\left(\begin{array}{cc} C & D\\ E & H \end{array}\right).$$

The matrices **C**, **D**, **E** and **H** are specified in electronic supplementary material, §S1.2 and S1.3. In brief, **C** is the community matrix and contains ecological variables, and **H** contains epidemiological variables [7]. At an infection-free steady state **E** = **0**. An infection-free steady state is **ecologically stable** if all eigenvalues of **C** have negative real part, *s*(**C**) < 0, where *s*(**C**) is the maximum real part of the eigenvalues of the matrix **C**. An infection-free steady state is **epidemiologically stable** if *s*(**H**) < 0.

The NGM is

$$K(N_{r},N_{g})=\left(\begin{array}{cc} \beta_{r}N_{r}T_{r} & \beta_{rg}N_{r}T_{g}\\ \beta_{gr}N_{g}T_{r} & \beta_{g}N_{g}T_{g} \end{array}\right),$$

evaluated at infection-free steady-state values of *N*_*r*_ and *N*_*g*_, where *T*_*r*_ and *T*_*g*_ are the expected times that infected red and grey squirrels (respectively) remain infectious. The NGM can be interpreted as follows. An infected red squirrel is infectious for an expected time *T*_*r*_ = 1/(*ν*_*r*_ + *α*), during which it infects other red squirrels at the rate *β*_*r*_ and grey squirrels at the rate *β*_*gr*_. An infected grey squirrel is initially infectious for an expected time *t*_*g*_ = 1/(*ν*_*g*_ + *σ*), during which a proportion *σt*_*g*_ enter the non-infectious compartment, *A*_*g*_. A proportion *p* = *η*/(*ν*_*g*_ + *η*) of these return to the *I*_*g*_ compartment, remain infectious for a further expected time *t*_*g*_, then a proportion *σt*_*g*_ of those return to the *A*_*g*_ compartment, and so on. Hence the total expected time that an infected grey squirrel is infectious is

$$T_{g}=t_{g}\sum_{\;j=0}^{\infty }{\left(\sigma t_{g}p\right)}^{\;j}=\frac{\nu_{g}+\eta }{(\nu_{g}+\eta )(\nu_{g}+\sigma )-\sigma \eta }.$$

During this time it infects other grey squirrels at the rate *β*_*g*_ and red squirrels at the rate *β*_*rg*_. An infection-free steady state is epidemiologically stable if *ρ*(**K**) < 1, where *ρ*(**K**) is the maximum eigenvalue of the matrix **K**(*N*_*r*_, *N*_*g*_) evaluated at the corresponding infection-free steady state. The matrix **H** may be decomposed H=T+ Σ, where **T** is a transmission matrix and  Σ is a transition matrix [7]. The NGM of large domain $K_{L}=-T{\;\Sigma }^{-1}$ has the same non-zero eigenvalues as **K** (see [41]). Hence the condition *ρ*(**K**) < 1 is equivalent to the condition *s*(**H**) < 0.

**Figure 1.**
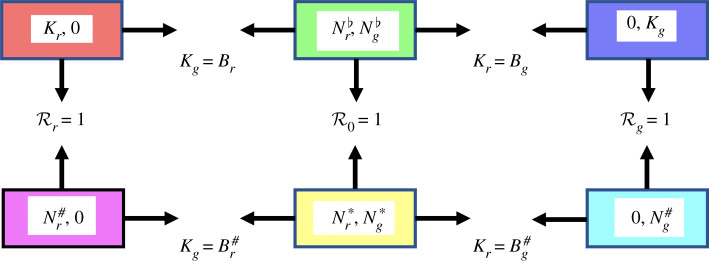
Summary of the three uninfected non-trivial steady states and three infected steady states, showing the thresholds for transition between them. The states are labelled with the steady-state values of *N*_*r*_ and *N*_*g*_ only. For further details, see table 2.

The analysis below, with details in electronic supplementary material, reveals that the system specified by equations (2.1) and (2.2) (with a fixed constant value of *N*_*p*_) has three non-trivial infection-free steady states, and three infected steady states. The steady states and the transitions between them are summarized in figure 1. Details of the transition thresholds are in table 2. In particular, we define three reproduction numbers for the onset of SQPV: $R_{r}=\beta_{r}K_{r}T_{r}$ where red squirrels only are present; $R_{g}=\beta_{g}K_{g}T_{g}$ where grey squirrels only are present; and $R_{0}=\rho (K(N_{r}^{♭},N_{g}^{♭}))$ where both squirrel species are present.

### 2.1. Infection-free steady states

In the absence of the predator and the pathogen (setting *N*_*p*_ = 0 and *I*_*r*_ = 0 in equation (2.1)), there are three non-trivial steady states. This could reflect the situation before the introduction of SQPV into the UK, and in areas where SQPV has not yet invaded and intra- and interspecies competition is mediated by the quality and suitability of the habitat. Factors such as the availability of food and nesting opportunities and the presence of enemies are assumed to determine the carrying capacity that each species could achieve in isolation. We explore differences in the outcome of competition in relation to differences in habitat suitability.

The steady state with red squirrels only, at carrying capacity (*N*_*r*_, *N*_*g*_) = (*K*_*r*_, 0), is stable if *K*_*g*_ < *B*_*r*_ (see table 2 and figure 1; electronic supplementary material, §S1.2). In other words, an ecosystem with red squirrels but no grey squirrels is unstable to invasion by grey squirrels if their carrying capacity exceeds the threshold *K*_*g*_ = *B*_*r*_. Similarly, the grey squirrel-only steady state (0, *K*_*g*_) is stable if *K*_*r*_ < *B*_*g*_. When the two species coexist, their steady-state population densities are

$$N_{r}^{♭}=\frac{\phi_{g}(\phi_{r}K_{r}-\phi_{rg}K_{g})}{\phi_{r}\phi_{g}-\phi_{rg}\phi_{gr}}\;\mathrm{and}\;N_{g}^{♭}=\frac{\phi_{r}(\phi_{g}K_{g}-\phi_{gr}K_{r})}{\phi_{r}\phi_{g}-\phi_{rg}\phi_{gr}}.$$

The two species can coexist if *ϕ*_*r*_*K*_*r*_ > *ϕ*_*rg*_*K*_*g*_ and *ϕ*_*g*_*K*_*g*_ > *ϕ*_*gr*_ *K*_*r*_, which can only be true if *ϕ*_*r*_*ϕ*_*g*_ > *ϕ*_*rg*_*ϕ*_*gr*_. In this context, existence means that the equations have a strictly positive solution. The coexistent steady state is stable whenever it exists. Here we are assuming that SQPV is not present in the squirrel populations. If SQPV were introduced, then the virus could invade the red squirrel-only steady state if $R_{r}>1$, invade the grey squirrel-only steady state if $R_{g}>1$, or invade the coexistent steady state if $R_{0}>1$.

**Figure 2.**
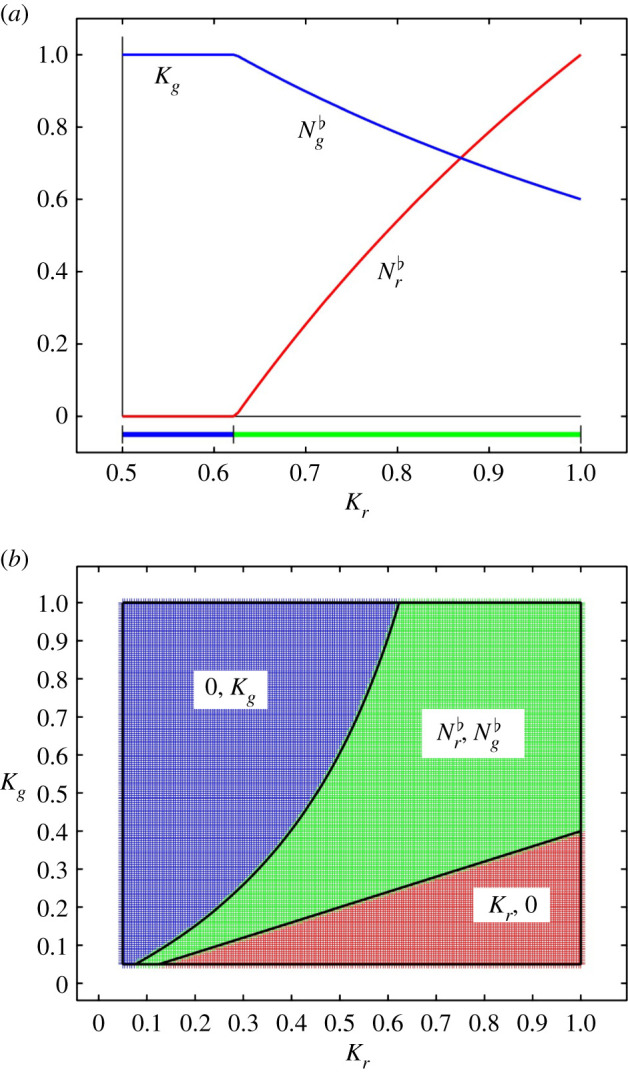
Competition between red and grey squirrels in the absence of SQPV. (*a*) Bifurcation diagram showing the stable steady states as functions of the red squirrel carrying capacity, *K*_*r*_, with the grey squirrel carrying capacity, *K*_*g*_ = 1. (*b*) the regions in the (*K*_*r*_, *K*_*g*_) plane where each steady state is stable. Colours in the lower bar (top) and plane bottom are red: red squirrels only; blue: grey squirrels only; green: red and grey squirrels.

We illustrate the interaction between the two species of squirrel by calculating their steady states for all values of their carrying capacities. For this, and without loss of generality, we chose the values of *ϕ*_*r*_ and *ϕ*_*g*_ to scale the carrying capacities of both squirrel species to one. We then simulated the degradation of the habitat suitability for red squirrels by replacing *ϕ*_*r*_ with *kϕ*_*r*_, hence *k* = *K*_*g*_/*K*_*r*_, and where two species are present, replacing *ϕ*_*rg*_ with (*k* − 1)*ϕ*_*rg*_. We require *k* ≥ 1, the assumption being that the carrying capacity for red squirrels is at best equal to that of grey squirrels. Parameter values are summarized in table 1. The bifurcation diagram (figure 2*a*) shows the steady states of both squirrel species as functions of the red squirrel carrying capacity. The idea behind the bifurcation diagram is that local differences may have evolved on a slow time scale, negatively affecting the suitability of the habitat for red squirrels compared to grey squirrels. This can occur as a result of ‘squirrel-independent’ decisions in forest management about tree composition (see [36] and references given there), for example by replanting with tree species that produce seeds that can be consumed less easily by red squirrels. This demonstrates how slow changes in the trophic level below the herbivore level of the squirrels qualitatively affect the herbivore populations. Figure 2*a* shows that, in the absence of SQPV, in habitats where the suitability for red squirrels is less than approximately 62% of grey squirrel carrying capacity, the grey squirrel replaces the red squirrel as a result of ecological competition only. In this simplified system, even a small decrease in suitability of the habitat for red squirrels leads to a situation where the grey squirrels outcompete the red squirrels and establish at higher population densities.

In figure 2*b*, we map the (*K*_*r*_, *K*_*g*_) plane, showing which steady states of the two species are stable as both of their carrying capacities vary. This summarizes the outcome of red and grey squirrel interaction as a result of competition only, for a large range of local habitats defined by their suitability for both species. Such an analysis may help to explain different local outcomes of red and grey squirrel interaction over a long period of cohabitation even before the introduction of SQPV into the ecosystem. Regardless of when or how often the virus was introduced into the UK, it is relevant that the model shows that even without SQPV grey squirrels are likely to outcompete red squirrels in their natural habitat (and that changes in the natural habitat can have strengthened that). The virus will further strengthen that decline, but there are parameter regions (i.e. circumstances) where the virus is not required to explain the decline. To what extent such parameter regions reflect naturally occurring circumstances is not the aim of our exploration. We suggest, however, that the range of dynamic situations and outcomes is more complex when one takes a broader view of relevant interactions, including the interaction with a pathogen.

Another way of looking at figure 2*b* is to interpret a reduction in *K*_*g*_ not as a decrease in habitat suitability but as a result of an increased death rate, *μ*_*g*_, for example because of local culling of grey squirrels. We see already from our simple model that the same system has a range of different outcomes depending on the background habitat suitability against which competition plays out. In recent years, the recognition of the importance of the local resources for red squirrel populations for their chances of survival has led to forest management scenarios aimed at favouring red squirrels, for example in Scotland and Wales [35,36]. In our bifurcation diagram, this would mean moving slowly to the right (higher values of *K*_*r*_), allowing for a broad range where stable coexistence is possible in the absence of other factors. In the next section, we explore this in greater detail, after we see how the picture changes when some of these other factors are introduced.

### 2.2. Infected steady states

We now examine the situation where SQPV has been introduced into the ecosystem, but maintain the absence of a (differentiating and explicit) predator. As well as the three infection-free non-trivial steady states already discussed, we have three infected steady states. With red squirrels only present, if $R_{r}>1$ we have $N_{r}=N_{r}^{\#}$ and $I_{r}=I_{r}^{\#}$ with *N*_*g*_ = *I*_*g*_ = *A*_*g*_ = 0. The steady state is unstable to invasion by grey squirrels if $K_{g}>B_{r}^{\#}$. The threshold $B_{r}^{\#}<B_{r}$; see table 2. With grey squirrels only present, if $R_{g}>1$ we have $N_{g}=N_{g}^{\#}=K_{g}$, $I_{g}=I_{g}^{\#}$, $A_{g}=A_{g}^{\#}$, with *N*_*r*_ = *I*_*r*_ = 0. The steady state is unstable to invasion by red squirrels if $K_{r}>B_{g}^{\#}$. The threshold $B_{g}^{\#}>B_{g}$; see table 2. With red and grey squirrels present and SQPV, the steady-state values *N*_*r*_ = *N*_r_*, *I*_*r*_ = *I*_r_*, *N*_*g*_ = *N*_g_*, *I*_*g*_ = *I*_g_* and *A*_*g*_ = *A*_g_*, may be found numerically (for further details see electronic supplementary material, §S1.4). The thresholds for transition between steady states are summarized in table 2.

**Figure 3.**
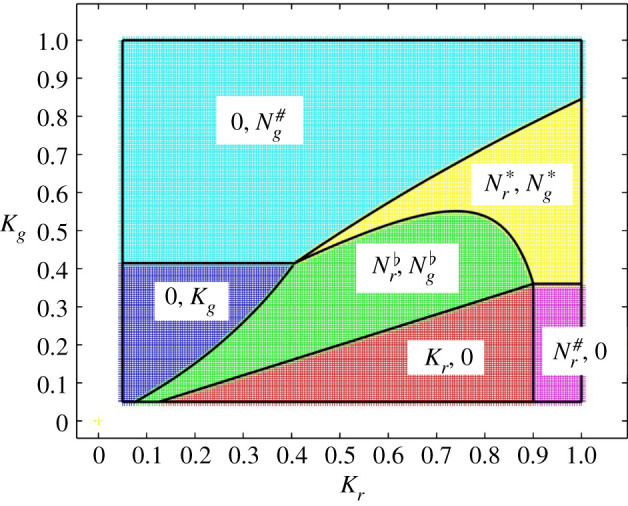
Competition between red and grey squirrels in the presence of SQPV. The figure shows the regions in the (*K*_*r*_, *K*_*g*_) plane where each steady state is stable. The colour key is red: red squirrels only; magenta: red squirrels and SQPV; blue: grey squirrels only; cyan: grey squirrels and SQPV; green: red and grey squirrels; yellow: red and grey squirrels and SQPV. The boundaries between the regions shown as black lines were calculated independently from *K*_*g*_ = *B*_*r*_, *K*_*r*_ = *B*_*g*_, $K_{g}=B_{r}^{\#}$, $K_{r}=B_{g}^{\#}$, $R_{r}=1$, $R_{g}=1$ and $R_{0}=1$.

**Figure 4.**
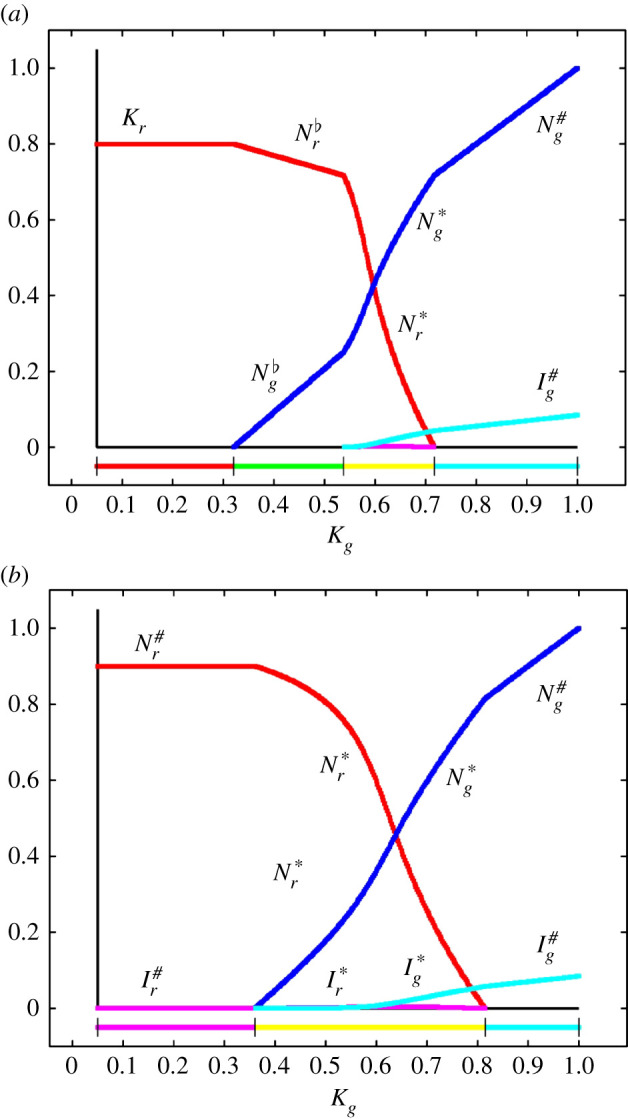
Competition between red and grey squirrels in the presence of SQPV. Bifurcation diagrams showing the stable steady states as functions of the grey squirrel carrying capacity *K*_*g*_. (*a*) the red squirrel carrying capacity *K*_*r*_ = 0.8; (*b*) *K*_*r*_ = 0.95. Colours in the lower bars denote stable steady states—red: red squirrels only; magenta: red squirrels and SQPV; cyan: grey squirrels and SQPV; green: red and grey squirrels; yellow: red and grey squirrels and SQPV.

In figure 3, the regions in the (*K*_*r*_, *K*_*g*_) plane where the different steady states are stable are shown. In figure 4, two bifurcation diagrams showing the steady states as a function of *K*_*g*_ with fixed values of *K*_*r*_ are presented, corresponding to vertical transects through the map in figure 3. The observations are similar to those of §2.1 but with a richer set of potential outcomes for the interaction.

It can be seen that in England, in the absence of the predator, it would have taken only a small reduction in the carrying capacity of red squirrels for the introduction of grey squirrels, together with SQPV, to be sufficient to drive the red squirrel population to extinction. For example, with *K*_*r*_ = 0.8, *K*_*g*_ = 1.0 and in the absence of SQPV, figure 2*a* shows coexistence of red and grey squirrels with $N_{r}^{♭}=0.541$ and $N_{g}^{♭}=0.784$. In the presence of SQPV, we have grey squirrels, but the red squirrel population is driven to extinction; see figure 3. Now consider if it would be possible to protect the red squirrel population by increasing the mortality of grey squirrels, perhaps by culling. The bifurcation diagram, figure 4*a*, shows that the population consists entirely of grey squirrels for the carrying capacity *K*_*g*_ above the value where $K_{r}=B_{g}^{\#}$, which is *K*_*g*_ = 0.717. There is coexistence of red and grey squirrels below this value, and above *K*_*g*_ = *B*_*r*_ = 0.320, with SQPV present when $R_{0}>1$. The basic reproduction number $R_{0}=1$ when *K*_*g*_ = 0.538. Comparing the bifurcation diagram figure 4*a* with figure 4*b*, where *K*_*r*_ = 0.95, we see that when the carrying capacity of red squirrels is at the higher value SQPV is present at all values of *K*_*g*_ in the range. There is coexistence of the species when *K*_*g*_ takes values below where $K_{r}=B_{g}^{\#}$, which is at *K*_*g*_ = 0.815, and above $K_{g}=B_{r}^{\#}=0.360$.

In Scotland, the squirrel population is mostly dominated by red squirrels, and there have been repeated invasions by SQPV [42]. There is the added factor of the pine marten, which predominantly predates on grey squirrels. We can interpret effects of different levels of additional background mortality due to the predator by looking at figures 3 and 4 and decreasing the value of *K*_*g*_ to traverse to regimes with different qualitative dynamics. Alternatively, we can model the ecosystem including full predator dynamics (see electronic supplementary material). The results of a combined lower carrying capacity for grey squirrels because of predation pressure (one trophic level up), with a higher carrying capacity for red squirrels because of forest improvement (one trophic level down), can lead to systems where red and grey squirrels coexist, both in the presence and absence of SQPV. We do not argue that coexistence is necessarily likely as the outcome under naturally occurring conditions. We show that there are circumstances (regions of parameter space) in which coexistence is possible, even with the virus and the predator present. Changing circumstances, for example in habitat suitability on a slower time scale or faster changes in predation pressure, can cause the system to move between steady states or render some potential states unreachable. Figures 3 and 4 show that even small changes can lead to profound qualitative changes in outcome.

It is possible that the predator has effects on grey and red squirrels that add to the direct effect of increased mortality. In the presence of an active predator, the squirrel species may experience stress in a *landscape of fear* to different degrees that may influence their competitive ability [17,43–46].

## 3. On reservoirs and dilution

We now address two further issues with regard to the eco-epidemiology of SQPV: ‘Is the grey squirrel a reservoir of infection for SQPV?’; and ‘Is there a dilution effect in this system?’

### 3.1. Is the grey squirrel a reservoir of infection for SQPV?

The question should be rephrased: ‘Under what circumstances are grey squirrels a reservoir for the infection of red squirrels with SQPV?’. If $R_{0}>1$ then red and grey squirrels form a maintenance community for SQPV. We have used $R_{0}$ to denote the basic reproduction number of SQPV in a community of red and grey squirrels, Ω={r,g}, and in the notation of [9] $R_{0}$ is the spectral radius of the NGM: $R_{0}(\{r,g\})=\rho (K(N_{r}^{♭},N_{g}^{♭}))$. Then, from the static viewpoint, red squirrels alone form a minimal maintenance community if $R_{\left\{r\right\}}=\beta_{r}N_{r}^{♭}T_{r}>1$, and grey squirrels form a minimal maintenance community if $R_{\left\{g\right\}}=\beta_{g}N_{g}^{♭}T_{g}>1$. If $R_{0}>1$ and $R_{\left\{g\right\}}>1$ but $R_{\left\{r\right\}}<1$ then grey squirrels are a reservoir of infection for red squirrels in the static sense. What is not taken into account in the analysis from the static viewpoint is that removing a species from the ecosystem changes the population dynamics of the other species. Now consider the dynamic viewpoint. With our simple two-species ecosystem, {*r*, *g*}, removing red squirrels would increase the population density of grey squirrels from $N_{g}^{♭}$ to *K*_*g*_, and removing grey squirrels would increase the population density of red squirrels from $N_{r}^{♭}$ to *K*_*r*_. The grey squirrel population can be regarded as a minimal maintenance community for SQPV if $R_{0}(\{g\})=R_{g}>1$, and similarly the red squirrel population can be regarded as a minimal maintenance community for SQPV if $R_{0}(\{r\})=R_{r}>1$. If $R_{g}>1$ and $R_{0}>1$ but $R_{r}<1$, then the grey squirrel is a reservoir of infection for SQPV in red squirrels in the dynamic sense.

**Figure 5.**
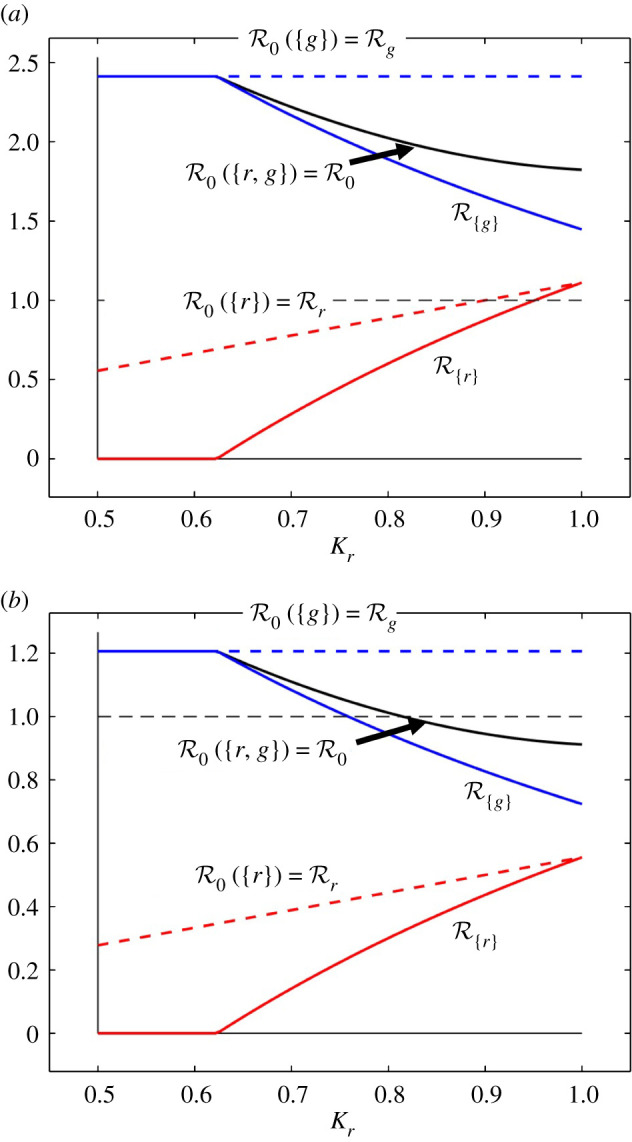
Reproduction numbers for SQPV as a function of the red squirrel carrying capacity *K*_*r*_, with grey squirrel carrying capacity *K*_*g*_ = 1. (*a*) Parameter values as in table 1; (*b*) parameter values as in table 1 except for *β*_*r*_ = 15 yr^−1^, *β*_*g*_ = 10 yr^−1^ and *β*_*rg*_ = *β*_*gr*_ = 5 yr^−1^. The values of $R_{\left\{r\right\}}$ and $R_{\left\{g\right\}}$ determine reservoir and maintenance status in the static sense, the values of $R_{r}$ and $R_{g}$ (broken curves) determine reservoir status in the dynamic sense. In both figures, there is a grey horizontal dashed line at the threshold value of one.

These concepts are illustrated in figure 5, where the reproduction numbers are presented as functions of the carrying capacity of red squirrels, *K*_*r*_, over the same range as that in figure 2*a*, and with *K*_*g*_ = 1. In figure 5*a*, it can be seen that red and grey squirrels form a maintenance community for SQPV, and grey squirrels form a minimal maintenance community for all values of *K*_*r*_ in the range. Red squirrels form a minimal maintenance community only for *K*_*r*_ > 0.951 in the static sense, or for *K*_*r*_ > 0.900 in the dynamic sense. If *K*_*r*_ < 0.900 then the density of the red squirrel population would be insufficient to support transmission of SQPV without the presence of the grey squirrel population. Hence the grey squirrel population is a reservoir of infection for the red squirrel population. If *K*_*r*_ > 0.951 then the red squirrel population density would be sufficient to support transmission of SQPV even if the grey squirrels were not present. Hence the grey squirrels should not be regarded as a reservoir of infection for the red squirrels. If 0.099 < *K*_*r*_ < 0.951, then the red squirrel population density would be insufficient to support transmission of SQPV if the grey squirrels were not present. However, removing the grey squirrels (somehow) would allow the red squirrel population to increase to a density that would support transmission. Hence in this region, the grey squirrel population is a reservoir of infection for the red squirrels in a static sense, but not in a dynamic sense.

In figure 5*b,* similar results are presented, but the values of all four infection rates (*β* values) are half of their values in table 1. The community of red and grey squirrels Ω={r,g} is a maintenance community for SQPV when $R_{0}>1$, which is now where *K*_*r*_ < 0.815. The red squirrel population does not form a maintenance community in either sense for any value of *K*_*r*_ in the range presented, and the grey squirrel population forms a minimal maintenance community in the static sense for *K*_*r*_ < 0.759, and in the dynamic sense throughout the range of *K*_*r*_. Hence the grey squirrel population may be regarded as a reservoir of infection for red squirrels in the static and dynamic sense for *K*_*r*_ < 0.815.

### 3.2. Is there a dilution effect in this system?

The results presented in figure 4 and described in the previous sections show that under most circumstances an increase in density of the grey squirrel population would lead to increased transmission of SQPV. If this translates as an increase in biodiversity leading to increased transmission we have the opposite of a dilution effect, an amplification effect. To formalize this, we find expressions for the elasticity of the steady-state prevalence of SQPV infection in red squirrels with respect to the population density of grey squirrels, $DY_{r}^{*}$, where $Y_{r}^{*}=I_{r}^{*}/N_{r}^{*}$; the elasticity of the abundance of infected red squirrels, $DI_{r}^{*}$; and the elasticity of the basic reproduction number, $DR_{0}$. Following [8], we calculate

$$DY_{r}^{*}=\frac{N_{g}^{*}}{Y_{r}^{*}}\frac{dY_{r}^{*}}{dN_{g}^{*}}=\frac{N_{g}^{*}}{Y_{r}^{*}}\frac{dY_{r}^{*}}{dK_{g}}/\frac{dN_{g}^{*}}{dK_{g}}.$$

The elasticity of the prevalence of infection in red squirrels is defined by its rate of change with respect to the grey squirrel population density, multiplied by $N_{g}^{*}/Y_{r}^{*}$ to make the quantity dimensionless. The calculation is facilitated by treating the carrying capacity *K*_*g*_ as the independent variable. The elasticity of the abundance of infected red squirrels is found by adding the elasticity of the red squirrel population density to the elasticity of the prevalence.

$$DI_{r}^{*}=DY_{r}^{*}+DN_{r}^{*}=\frac{N_{g}^{*}}{Y_{r}^{*}}\frac{dY_{r}^{*}}{dK_{g}}/\frac{dN_{g}^{*}}{dK_{g}}+\frac{N_{g}^{*}}{N_{r}^{*}}\frac{dN_{r}^{*}}{dK_{g}}/\frac{dN_{g}^{*}}{dK_{g}}.$$

To calculate the elasticity of the basic reproduction number, observe that $R_{0}=\rho (K(N_{r}^{♭},N_{g}^{♭}))$. Hence we need to find the elasticity of $R_{0}$ with respect to the infection-free steady-state density of grey squirrels. We define

$$DR_{0}=\frac{N_{g}^{♭}}{R_{0}}\frac{dR_{0}}{dN_{g}^{♭}}=\frac{N_{g}^{♭}}{R_{0}}\frac{dR_{0}}{dK_{g}}/\frac{dN_{g}^{♭}}{dK_{g}}.$$

For full details of the calculations see electronic supplementary material, §S2.

**Figure 6.**
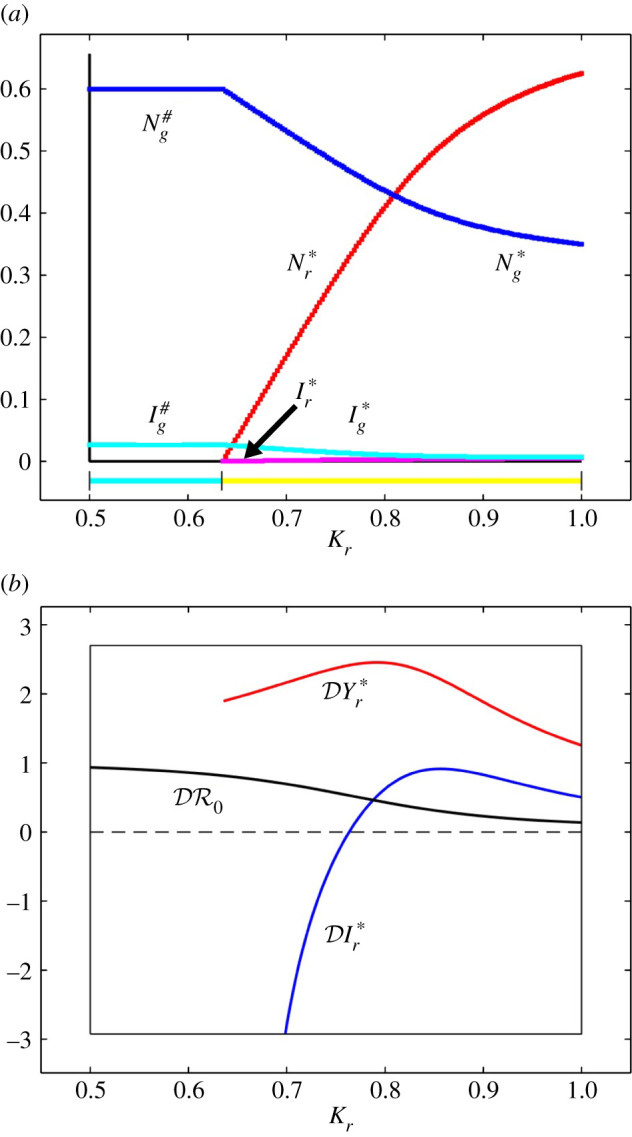
(*a*) The stable steady-state values of red and grey squirrels as functions of the red squirrel carrying capacity *K*_*r*_. (*b*) The elasticity of the steady-state prevalence of infection in red squirrels, $DY_{r}^{*}$, abundance of infection in red squirrels, $DI_{r}^{*}$, and basic reproduction number of SQPV, $DR_{0}$ as functions of *K*_*r*_. Parameter values as in table 1 except *μ*_*g*_ = 0.72 yr^−1^ leading to *K*_*g*_ = 0.6. Colours in the lower bar (*a*) denote stable steady states—cyan: grey squirrels and SQPV; yellow: red and grey squirrels and SQPV.

In figure 6*a,* we present the stable steady-state values of *N*_*r*_, *N*_*g*_, *I*_*r*_ and *I*_*g*_, for 0.5 ≤ *K*_*r*_ ≤ 1.0 and *K*_*g*_ = 0.6. The elasticities $DY_{r}^{*}$, $DI_{r}^{*}$ and $DR_{0}$ are presented for the same values of *K*_*r*_ and *K*_*g*_ in figure 6*b* ($DY_{r}^{*}$ and $DI_{r}^{*}$ where $I_{r}^{*}>0$). It can be seen that for all values of *K*_*r*_ the prevalence of infection in red squirrels increases with the grey squirrel population density, $DY_{r}^{*}>0$, which represents an amplification effect. As increasing the abundance of grey squirrels decreases the abundance of red squirrels, it is not surprising that at least for some values of *K*_*r*_ we have $DI_{r}^{*}<0$, corresponding to a dilution effect. Figure 6*b* also shows that for all values of *K*_*r*_ in the range, $DR_{0}>0$, corresponding to an amplification effect.

Now consider the role played by the predator. We have assumed that predator population densities are maintained at equilibrium with $N_{\;p}=N_{\;p}^{*}$ (for a full dynamic analysis see electronic supplementary material), and defined the grey squirrel carrying capacity by $K_{gp}=(\nu_{g}-\mu_{g}-$ $\psi_{gp}N_{\;p}^{*})/\phi_{g}$. To define the elasticities of the prevalence and abundance of SQPV infection in red squirrels and of $R_{0}$ with regard to predator population density, we need to multiply the elasticities defined above by

$$\frac{N_{\;p}^{*}}{N_{g}^{*}}\frac{dN_{g}^{*}}{dK_{g}}/\frac{dN_{\;p}^{*}}{dK_{g}}=-\frac{\psi_{gp}N_{\;p}^{*}}{\phi_{g}N_{g}^{*}}\frac{dN_{g}^{*}}{dK_{g}},$$

and hence reversing the sign of the expression. Hence a positive elasticity with regard to grey squirrel population density (amplification) corresponds to a negative elasticity with regard to predator population density (dilution).

## 4. Discussion

We have presented and analysed a model for the interaction of two herbivore species competing for resources, in the presence of a pathogen infecting both of them, and in response to changes in the trophic levels below (resource) and above (predator). We showed, in an example system, that the specifics of the ecological interactions at the trophic level of the host species and between the directly related trophic levels strongly influence the epidemiological dynamics. This influence is most pronounced if there are differential effects in various processes (particularly: competition affecting one species more than the other, differences in utilization of the resources by the two host species, one host species being more affected by the pathogen than the other, differences in the predation pressure experienced by the two host species). Disentangling the concepts of ecological and epidemiological stability of the model system is relevant for insight into ecosystem behaviour, relating to the possibilities of being invaded by another species or a pathogen, respectively. We focussed on invasion success ($R_{0}$), on the capacity of a host species to act as a maintenance host or a reservoir of infection towards a target host, and on the question whether or not (and in what sense) a dilution effect may operate in the system. All these aspects depend on the ecological context in which the host species and pathogen occur in their natural setting (see also the perspective by [3]). In earlier work, we have shown this in a non-specific general setting of *n* interacting species, some of which are hosts to a particular pathogen [7–9].

Our analysis implies that when studying the infection dynamics of a given pathogen in a natural system, it is necessary to include ecological interactions between a range of species that is broader than the community of host species of that pathogen, and to take an ecosystem view including other species with which the host species interact on different trophic levels. Natural events, changing environmental processes, human influence through conservation and nature management decisions, harvesting, and destruction or changes to habitat can affect different (groups of) species in an ecosystem in different ways. Ecological and epidemiological interactions between species can have an indirect, unintended and even counter-productive impact on host species.

We explored these interactions in a specific natural system that has received substantial attention in the literature because of the important conservation-related issues involved. It was not our intention to improve on existing modelling work for this system, or to provide quantitative analyses or predictions to help conservation efforts and decisions in this complex area. We focused primarily on steady states of the dynamical system. Our aim was to show in a *real-life* system how ecological and epidemiological processes and mechanisms influence each other, and how they collectively determine which of the many steady states can be attained and under which conditions. We also showed how changes in the underlying processes and the strengths of various interactions determine changes in the steady state in which the system can be expected to settle. Other factors that were not considered but may impact on the ecological and epidemiological processes of our system are, for example, the so-called *landscape of fear* [43,44] (the observation of newly emerging predation pressure-induced stress in prey species leading to changing behaviour) and sub-clinical effects on host species that may influence their ecological interactions (for example through subtle changes in consumer–resource interaction, both as consumer and as resource). These factors could be regarded as implicitly present in the model, through changes in the squirrel carrying capacity.

Despite the fact that we in no way aimed to present a detailed model of the squirrel system, there are a number of potentially relevant observations to be made from our qualitative analysis. If grey squirrels are the superior species in competition with red squirrels, then in the absence of a differentiating predator that favours grey squirrels and in the absence of SQPV, grey squirrels are likely to eventually outcompete red squirrels for a large range of parameter values describing their interaction. A gradual change of resource quality (tree type dictating the type of seeds and nuts available) favouring grey squirrels over red squirrels will allow them to exploit this range. We chose not to model the dynamics of the food quality explicitly. For our analysis that seems a reasonable choice because the time scale for forest change (structure, yield and composition) is slow compared to the squirrel life span. Hence we assumed there to be no substantial or essential feedback from squirrels to forest composition. Grey squirrels have been observed to damage a variety of tree species including oak through ‘bark stripping’ [31], and this could be included as a feedback mechanism in future studies. The introduction into this system of a differentiating virus that kills red squirrels but does not negatively affect grey squirrels, decreases the possibility for coexistence even further and makes the outcome of a grey squirrel-only steady state virtually inevitable.

One way to prevent the outcome of a stable grey squirrel-only steady state is to maintain an additional pressure on grey squirrels, for example, in the form of an increased death rate. This can be achieved by culling, or by introducing a predator that favours grey squirrels over red squirrels. The pine marten may play this latter role in Scotland and its influence on the grey squirrels increases the region of parameter space where grey and red squirrels can coexist, and can even lead to a red squirrel-only steady state when predation pressure is sufficiently high. A change in habitat and food quality specifically aimed at favouring red squirrels (in contrast to what we discussed above where these changes were made independent of squirrel-related considerations) may further increase the region where the red squirrel population can be maintained in a stable way.

It is important to determine how a shift in forest structure and composition can be used to strengthen the competitive ability of red squirrels in areas where the species is still abundant, where grey squirrels are just being introduced, the virus has not yet successfully invaded and pine martens as predators are on the rise. Forest management may be able to push the system to a region where red squirrels can survive in this broader eco-epidemiological context [10,32,36]. To assess such scenarios in a qualitative analysis, we can no longer assume that food quality is constant on the squirrel time scale or that squirrels have no influence on it. For forest improvement favouring red squirrels, there is direct feedback from squirrel abundance to human-induced habitat change. What should be clear from our analysis is that one should not neglect the other aspects of the system (i.e. differentiating predation from the carnivore trophic level, incursions of SQPV and uneven competition between the squirrel species) as the outlook for red squirrel-induced forest improvement may differ depending on these aspects.

We have also shown that in general, the ecosystem context determines whether a (group of) host species can be considered a reservoir of infection for a given pathogen towards a specific target host species [9]. The same (group of) host species may be a reservoir in one ecosystem context but not in another. We illustrated this issue in the case of grey squirrels using our simple model. It depends on the circumstances whether the grey squirrel can act as a reservoir of infection with SQPV for the red squirrel, whether the red squirrel could maintain the pathogen in isolation, or whether both species are needed as a maintenance community.

In earlier work [8], we showed that it matters how we quantify dilution/amplification to investigate whether or not a system shows a dilution/amplification effect with respect to a given pathogen and changes in system biodiversity. Our analysis of the squirrel system highlights an additional issue, i.e. it matters how we measure biodiversity. There is a rich literature on quantifying biodiversity, detailing what to measure and how to translate this information into meaningful indices [47,48]. Concepts such as species richness, species density/abundance and evenness are relevant. Our toy example, with a limited view of a real system, shows that the choice of abundance or richness influences the conclusions. This suggests a need to study the interaction between pathogens and biodiversity using a variety of measures for biodiversity and in larger food web/ecosystem-contexts.

In conclusion, we have used a qualitative mathematical model to demonstrate how ecology and epidemiology interact in determining the dynamics of infectious diseases. Although we have presented our arguments in terms of the population dynamics of red and grey squirrels, infection with SQPV and the influence of a predator, our results are not specific to this system. Our aim has always been to derive criteria that can be applied to eco-epidemiological dynamics in a wide variety of situations. Understanding the epidemiology of infections of wild animals is of increased importance, especially where the pathogen has zoonotic potential.

## Data accessibility

This article has no additional data.
